# Editorial: Integrative Toxicogenomics: Analytical Strategies to Amalgamate Exposure Effects With Genomic Sciences

**DOI:** 10.3389/fgene.2018.00563

**Published:** 2018-11-27

**Authors:** Pierre R. Bushel, Weida Tong

**Affiliations:** ^1^Microarray and Genome Informatics Group, Biostatistics and Computational Biology Branch, National Institute of Environmental Health Sciences, >Durham, NC, United States; ^2^Division of Bioinformatics and Biostatistics, National Center for Toxicological Research, U.S. Food and Drug Administration, Jefferson, AR, United States

**Keywords:** toxicogenomics, gene expression, toxicology, chemicals, mode of action, integration, bioinformatics, gene regulatory network

Since the advent of toxicogenomics (TGx; Afshari et al., 1999; Nuwaysir et al., 1999), the expectation of its foreseeable impact to risk/safety assessment has given way to the reality that there is much that still needs to be done to realize its full potential. One of the advancements is in the area of integrative TGx which is focused on amalgamating (i.e., merging) diverse data resources with TGx data to gain a comprehensive understanding of toxicology. This requires innovation in bioinformatics, statistics and systems toxicology and typically a combination of the utility of two of more of these disciplines. In addition, integration of data takes place at one of three stages (Bushel, 2016): (1) early—combining separate data into a single matrix and analyzing it, (2) intermediate—keeping the data separate and applying a separate analytic function to each followed by combining of the functions or (3) late—analyzing each data set separately and then merging the results. This Frontiers in Toxicogenomics Research Topic focuses on integrative TGx at the late stage and brings together analyses that combine gene expression (microarray, TempO-Seq or RNA-Seq) with other data (biological assays, clinical chemistry, therapeutic categories or molecular pathways) or highlights data analytics that leverage bioinformatics and statistics. The eight articles illustrate the state-of-art in the field and the amalgamation of TGx data or incorporation of analytical methodologies for a more comprehensive deduction of biological mechanisms and cellular functions associated with adverse outcomes from environmental exposures and toxicants. However, it is clear that the field of integrative TGx needs considerably more attention paid to the merging and analysis of the data at the early and intermediate stages.

Dose-response and time-dependency are key features in toxicology. Nowadays, TGx study designs can easily incorporate at least one of these two features to assess gene expression-based point-of-departure and/or early biomarkers. Zhang et al. assess the toxic effect of doses of Zearalenone on cultured donkey granulosa cells (dGCs) by interrogating gene expression data from RNA sequence (RNA-Seq) analysis and visualization of apoptosis through a tunnel assay. The integrative analysis of the gene expression data, RT-qPCR and immunofluorescence staining of dGCs supports the dysregulation of apoptosis-related genes and induction of ovarian cancer-related genes via the PTEN/PI3K/AKT signaling pathway. Liu et al. leverage a previously developed methodology called pair ranking (PRank) to compare three preclinical rat TGx microarray data sets for assessment of *in vitro* to *in vivo* extrapolation. They show that there was a high degree of agreement between the *in vivo* assay systems (24 h and 28 days) and similarity between the *in vitro* and the 28 days *in vivo* systems suggesting that a short-term *in vivo* assay system might be practical for some endpoints in order to save time and resources for drug safety evaluation and risk assessment. In addition, Souza et al. reverse engineer gene regulatory networks using *in vitro* human microarray gene expression data to reveal dose-dependent, chemical-specific mechanisms of action in stress-related biological networks. Although gene expression microarrays and RNA-Seq have a solid presence in certain areas of application, TGx studies have begun to explore targeted sequencing using the templated oligo sequencing detection assay (TempO-Seq™). House et al. report a bioinformatics pipeline to analyze dose-response gene expression data profiled with TempO-Seq in induced pluripotent stem cell-based cardiomyocytes. TempO-Seq has drawn significant attention in the TGx community due to its low cost, high throughput and easy to operate.

One of the most extensive applications of TGx is to gain an improved understanding of underlying mechanisms of treatment. While molecular initiative events for many chemicals are well-studied, their modes-of-action (MOAs) remain to be determined. Using RNA-Seq data for the aforementioned purpose can be challenging given the inherent noise. Lozoya et al. presents a leveraged signal-to-noise ratio (LSTNR) thresholding method to identify differentially expressed genes and reveal gene expression patterns especially from samples with very few replicates. Using their method to analyze RNA-Seq rat liver data generated through the MicroArray Quality Control phase III (MAQC3) SEquence Quality Control (SEQC) TGx study, they show that many of the chemicals cluster by MOA and that there are several genes that appear to function as biomarkers specific for chemicals with similar MOA. In addition, Hawliczek-Ignarski et al. investigate whether TGx profiles are able to group chemicals by MOA. As a proof-of-concept study, they tested the hypothesis with data generated after *in vitro* exposure of an established cell line to group chemicals with an uncoupling MOA. Furthermore, Funderburk et al. describe a weighted network analysis of rat liver gene expression from *in vivo* studies involving chemicals from several known MOAs (SEQC TGx study). They demonstrate that overlaps in toxicologic pathways by chemicals with different MOAs (receptor-mediated vs. non-receptor-mediated) reveal points of potential crosstalk between regulatory pathways.

Like mRNAs, microRNAs (short, non-coding RNA molecules roughly 22 nucleotides) are important in toxicology research as well because they are known to regulated gene expression (Bartel, 2004). Bisgin et al. evaluate bioinformatic tools and parameters for RNA-Seq analysis of microRNAs from the livers of rats exposed to thioacetamide at multiple doses and time points. They conclude that variation in the hairpin loop of microRNAs is small relative to the treatment effect and that normalization of the data introduced a large variation in differentially expressed microRNAs. In addition, they indicate that the miRDeep2 analysis tool was preferable over the other choices.

From this small collection of articles, the power and promise of integrative toxicogenomics may seem reassuring. The desire is that as toxicogenomic studies broaden in scope and design, and the complexity of the data increases exponentially, new cutting-edge research and development of novel methodologies to manage, integrate, and analyze massive amounts of data will continue to evolve.

## Author contributions

All authors listed have made a substantial, direct and intellectual contribution to the work, and approved it for publication.

### Conflict of interest statement

The authors declare that the research was conducted in the absence of any commercial or financial relationships that could be construed as a potential conflict of interest.
